# Do chromosome rearrangements fix by genetic drift or natural selection? Insights from *Brenthis* butterflies

**DOI:** 10.1111/mec.17146

**Published:** 2023-10-09

**Authors:** Alexander Mackintosh, Roger Vila, Simon H. Martin, Derek Setter, Konrad Lohse

**Affiliations:** ^1^ Institute of Ecology and Evolution University of Edinburgh Edinburgh UK; ^2^ Institut de Biologia Evolutiva (CSIC‐Universitat Pompeu Fabra) Barcelona Spain

**Keywords:** genomics/proteomics, inbreeding, insects, molecular evolution, natural selection and contemporary evolution, population genetics ‐ empirical

## Abstract

Large‐scale chromosome rearrangements, such as fissions and fusions, are a common feature of eukaryote evolution. They can have considerable influence on the evolution of populations, yet it remains unclear exactly how rearrangements become established and eventually fix. Rearrangements could fix by genetic drift if they are weakly deleterious or neutral, or they may instead be favoured by positive natural selection. Here, we compare genome assemblies of three closely related *Brenthis* butterfly species and characterize a complex history of fission and fusion rearrangements. An inferred demographic history of these species suggests that rearrangements became fixed in populations with large long‐term effective size (*N*_e_), consistent with rearrangements being selectively neutral or only very weakly underdominant. Using a recently developed analytic framework for characterizing hard selective sweeps, we find that chromosome fusions are not enriched for evidence of past sweeps compared to other regions of the genome. Nonetheless, we do infer a strong and recent selective sweep around one chromosome fusion in the *B. daphne* genome. Our results suggest that rearrangements in these species likely have weak absolute fitness effects and fix by genetic drift. However, one putative selective sweep raises the possibility that natural selection may sometimes play a role in the fixation of chromosome fusions.

## 1 INTRODUCTION

### 1.1 How do chromosome rearrangements fix?

Eukaryotic genomes vary widely in chromosome number and structure, that is, karyotype. While closely related species often have similar karyotypes, there are also examples of considerable variation in chromosome number within genera (Hipp et al., 2009; Lukhtanov, 2015) and even species (John & Hewitt, 1970; Searle, 1991; Zima et al., 1996). This variation is typically generated through chromosome rearrangements, with chromosome fissions and fusions resulting in increases and decreases in chromosome number respectively. These rearrangements have been shown to promote speciation as well as influence the rate and distribution of recombination events (Bidau et al., 2001; Davey et al., 2017; Mackintosh et al., 2023; Näsvall et al., 2023; Yoshida et al., 2023), but our understanding of their role in evolution is limited by the fact that we do not know how they rise to high frequency in the first place. Heterozygosity for fissions or fusions can cause improper segregation during meiosis (White, 1973), and so it is often suggested that new rearrangements are weakly deleterious and establish through strong genetic drift (Bush et al., 1977; Wilson et al., 1975). An alternative view is that rearrangements become fixed because they are favoured by natural selection (Bickham & Baker, 1979; Qumsiyeh & Handal, 2022), but there is currently limited empirical evidence to support this.

There are a number of different ways for a fission or fusion to be advantageous. For example, a chromosome fusion can increase linkage disequilibrium between coadapted alleles, leading to enhanced local adaptation and fixation of the rearrangement (Charlesworth, 1983; Fisher, 1930; Guerrero & Kirkpatrick, 2014). There are examples of fused chromosomes that are enriched for adaptive loci (Liu et al., 2022; Wellband et al., 2019), but it is unclear what fraction of these variants predate the rearrangements and potentially contributed to their fixation. Rearrangements can also have direct effects on gene expression, either through changes in genome positioning within the nucleus (Di Stefano et al., 2020) or if breakpoints occur within a gene body or regulatory element (Harewood & Fraser, 2014). While most changes in gene expression are likely deleterious, any beneficial changes could lead to the spread of a rearrangement. Meiotic drive is another mechanism by which chromosome rearrangements could rapidly increase in frequency. This process involves drive alleles that are transmitted to gametes more than 50% of the time and typically occurs within asymmetric meiosis (Pardo‐Manuel de Villena & Sapienza, 2001b). Chromosome rearrangements with differences in centromere size or form can act as drive alleles which leads to their fixation (Pardo‐Manuel de Villena & Sapienza, 2001a; Stewart et al., 2019). Although this process is primarily associated with monocentric chromosomes (i.e. those with a single centromere), it has also been suggested to occur in organisms with holocentric chromosomes, such as nematodes and Lepidoptera, where centromeres are not localized (Bureš & Zedek, 2014).

While the processes described above are certainly possible, the fixation of fissions and fusions may not be adaptive at all. Instead, a rearrangement could fix entirely through genetic drift (Wright, 1941). This may be the case if meiosis is robust to the risk of unbalanced segregation associated with heterokaryotypes (Borodin et al., 2019). Even if a rearrangement does confer a fitness cost, strong drift and inbreeding in small populations could still lead to its fixation (Lande, 1979; Wright, 1941). Under this scenario, one would expect more rearrangements involving Y/W chromosomes than those involving X/Z chromosomes, due to the approximately threefold difference in effective population size (*N*_e_). Pennell et al. (2015) tested this prediction and found that Y/W‐autosome fusions are indeed significantly more common than X/Z‐autosome fusions in fish and squamate reptiles, though not in mammals. They, therefore, suggest that sex–autosome fusions are often weakly deleterious and fix through genetic drift. While the same could be true for fissions and autosome–autosome fusions, it is unclear whether all of these rearrangements have similar fitness effects.

### 1.2 Inferring selective sweeps

If fissions and fusions rise in frequency due to natural selection, sites that are tightly linked to recent rearrangements will show evidence of selective sweeps. This process, in which a beneficial allele increases rapidly in frequency and nearby alleles ‘hitchhike’ with it, leaves a signature in population genomic data that can be used to infer past selection (Maynard Smith & Haigh, 1974). A variety of methods have been developed for sweep inference, often making use of different types of genomic data, such as allele frequencies (Nielsen et al., 2005), patterns of haplotype similarity (Garud et al., 2015; Harris & DeGiorgio, 2020) or even reconstructed ancestral recombination graphs (Hejase et al., 2022; Stern et al., 2019). One limitation shared by a number of methods is the assumption that the modelled selective sweep has completed very recently. This limits the power to detect and accurately characterize even strong sweeps that happened deeper in time.

Recently, Bisschop et al. (2021) showed that, for small sample sizes, the joint distribution of genealogical branch lengths can be derived under an approximate model of a selective sweep. This allows the calculation of composite likelihoods from mutation configurations in short sequence blocks, in particular the blockwise site frequency spectrum (bSFS; Bunnefeld et al., 2015). Importantly, this analytic framework can be used to infer and characterize sweeps that happened further back in time (i.e. >0.1 *N*_e_ but <4 *N*_e_ generations ago) by treating the sweep as a discrete event that is both preceded and followed by a neutral coalescent process (Bisschop et al., 2021). This inference method can therefore be used to test whether natural selection has acted on certain regions of the genome, even if the selective events are relatively old.

### 1.3 Overview

Here, we use the fast rate of chromosome evolution in *Brenthis* fritillary butterflies to investigate how chromosome fissions and fusions evolve. Previous work has shown that chromosome numbers vary substantially among *Brenthis* species (Mackintosh et al., 2022; Pazhenkova & Lukhtanov, 2019; Saitoh, 1986, 1991; Saitoh & Lukhtanov, 1988) and that this variation is due to chromosome rearrangements (Mackintosh et al., 2023) rather than differences in ploidy or supernumerary chromosomes. The genus consists of four species, *B. daphne* (Denis and Schiffermüller, 1775), *B. ino* (Rottemburg, 1775), *B. hecate* (Denis and Schiffermüller, 1775) and *B. mofidii* (Wyatt, 1969), and here, we analyse genomic data from the first three. First, we describe a newly generated chromosome‐level genome assembly for *B. hecate*. This species has a much larger number of chromosomes (*n*_c_ = 34) than *B. daphne* (*n*_c_ = 12–13) or *B. ino* (*n*_c_ = 13–14), implying a history of rapid rearrangement. Second, we compare the genomes of these three *Brenthis* species with publicly available genome assemblies of two other fritillary butterfly species in the tribe Argynnini. Using a maximum parsimony method, we show that almost all rearrangements are confined to the genus *Brenthis*. Third, we use whole genome resequence data for all three *Brenthis* species to estimate their demographic history. This allows inferred rearrangements to be placed within the context of species divergence times and effective population sizes. Finally, we investigate whether chromosome fusions, the most common rearrangement type in our data set, have fixed through hard selective sweeps. For each of 12 potentially recent chromosome fusions, we use the analytical framework of Bisschop et al. (2021) to estimate support for a hard sweep model as well as the time since the sweep and the strength of selection.

## 2 MATERIALS AND METHODS

### 2.1 Sampling and sequencing

Butterflies were collected by hand netting and frozen from live in a −80 freezer. We performed a high molecular weight (HMW) DNA extraction for one *Brenthis hecate* individual (ES_BH_1412; Table S1) using a salting out protocol (see Mackintosh et al., 2022 for details). For four other *B. hecate* individuals (Table S1), DNA was extracted from Ethanol preserved samples using a Qiagen DNeasy Blood & Tissue kit, following the manufacturer's instructions. Edinburgh Genomics (EG) prepared TruSeq Nano gel free libraries from all five DNA extractions and sequenced them on an Illumina NovaSeq 6000. EG also generated a SMRTbell sequencing library from the HMW DNA and sequenced it on a Pacbio Sequel I instrument. A sixth individual (ES_BH_1411; Table S1) was used for chromatin conformation capture (HiC) sequencing. EG performed the HiC reaction using an Arima‐HiC kit, following the manufacturer's instructions for flash frozen animal tissue and generated a TruSeq library which was sequenced on an Illumina NovaSeq 6000.

### 2.2 Genome assembly

We generated a reference genome for *B. hecate* by assembling Pacbio continuous long reads with nextdenovo version 2.4.0 (Hu et al., 2023). The contig sequences were polished with Illumina short‐reads from the same individual using hapo‐g version 1.1 (Aury & Istace, 2021). We identified and removed haplotypic duplicates and contigs deriving from other organisms using purge_dups version 1.2.5 (Guan et al., 2020) and blobtools version 1.1.1 (Laetsch & Blaxter, 2017) respectively. We mapped HiC data to the contigs with bwa‐mem version 0.7.17 (Li, 2013) and then used yahs version 1.1a.2 and juicebox version 1.11.08 to scaffold the assembly into chromosome‐level sequences (Robinson et al., 2018; Zhou et al., 2023).

### 2.3 Synteny analysis

We compared synteny between five genome assemblies of butterfly species in the tribe Argynini: *Brenthis hecate*, *Brenthis ino* (Mackintosh et al., 2022), *Brenthis daphne* (Mackintosh et al., 2023), *Fabriciana adippe* (Lohse et al., 2022) and *Boloria selene* (Lohse et al., 2022). Pairwise alignment of assemblies was performed with minimap2 version 2.17 (Li, 2018) and differences in synteny were visualized by plotting high‐quality alignments (mapping quality of 60 and length >=50 kb). We found that the genome sequence of *B. selene* has low sequence identity to the other genomes, resulting in few nucleotide alignments. We therefore identified BUSCO genes in all five assemblies (lepidoptera_odb20, busco version 5.3.2, Simão et al., 2015) and used the location of these BUSCO genes to visualize synteny between the *B. selene* genome and the others.

We estimated the number of fission and fusion rearrangements across the phylogeny of these species using syngraph (https://github.com/A‐J‐F‐Mackintosh/syngraph). We included an additional nymphalid genome assembly in this analysis (*Nymphalis polychloros*, Lohse et al., 2021) as an outgroup. BUSCO genes were used as markers and the minimum number of markers for a rearrangement to be reported was set to five. We used the tabulated output of syngraph, as well as the paf files generated by minimap2, to identity approximate positions of chromosome fusion points.

### 2.4 Fitting a multispecies demographic history

To infer a demographic history for the three *Brenthis* species, we mapped whole genome resequencing (WGS) data to the *F. adippe* reference genome. This included data for five *B. hecate* individuals (Table S1), as well as seven *B. daphne* and six *B. ino* individuals that were originally analysed in Mackintosh et al. (2023). Individuals were sampled from across the Palaearctic (Table S1, see figure 1 in Mackintosh et al., 2023), including different glacial refugia.

WGS data were trimmed with fastp version 0.2.1 (Chen et al., 2018) and mapped with bwa‐mem. Variants were called with freebayes version 1.3.2 (Garrison & Marth, 2012) and filtered with gimble preprocess (Laetsch et al., 2022) using the following options: ‐‐snpgap 2 ‐‐min_qual 10 ‐‐min_depth 8 ‐‐max_depth 5. Here, ‐‐snpgap is the minimum distance a SNP can be from an indel, ‐‐min_qual is the minimum quality score of a SNP, ‐‐min_depth is the minimum absolute read depth and ‐‐max_depth is the maximum read depth relative to the sample‐specific mean. We applied an additional filter to remove SNPs where >70% of individuals were heterozygous, as these are likely due to alignment of paralogous sequence. We annotated genes in the *F. adippe* genome (see Supplementary Methods) and used this to restrict our analysis to fourfold degenerate (4D) sites.

Given 295,730 SNPs, as well as a total count of 4D sites callable across all individuals (2,487,949), we generated an unfolded three‐dimensional site frequency spectrum (3D‐SFS) using get_3D_SFS.py (see Data accessibility). The ancestral state at each SNP was assigned using the reference (*F. adippe*) allele. After inspection of the 3D‐SFS, we chose to fold the data due to an excess of high frequency‐derived alleles that likely represent polarization error.

Demographic modelling was performed with fastsimcoal2 (fsc27093; Excoffier et al., 2021). We fit a model of divergence with gene flow between the three *Brenthis* species which included two split times, six effective population sizes (*N*_e_) and eight asymmetrical effective migration rates (*m*_e_; 16 parameters total, Figure 2c; Table S2). Each *N*_e_ and *m*_e_ parameter within this model remains constant between speciation events. The parameter estimates with the greatest composite likelihood were recorded as point estimates, and we performed 100 parametric bootstraps to obtain 95% confidence intervals (95% CIs). The lower 95% CIs were calculated by interpolating between the second and third percentiles and the upper 95% CI was calculated by interpolating between the 97th and 98th. Demographic parameter estimates were scaled using a de novo mutation rate of 2.9 × 10^−9^ (Keightley et al., 2015). The fastsimcoal2 commands used are listed in the Supplementary Methods.

### 2.5 Identifying runs of homozygosity

We identified runs of homozygosity (ROH) in each *Brenthis* individual to gain more information about genetic drift in the recent past of these species. To do this, we mapped WGS data for each *Brenthis* species to the corresponding (species‐specific) reference genome with bwa‐mem. Variants were called within each species using freebayes and filtered with gimble preprocess: ‐‐snpgap 2 ‐‐min_qual 10 ‐‐min_depth 8 ‐‐max_depth 1.5 (see above for an explanation of these options). We restricted SNPs in the VCF to non‐repeat regions where all individuals had a genotype. We then identified runs of homozygosity (ROH) in each individual using plink version 1.90b6.18 (Purcell et al., 2007) with the following options: ‐‐homozyg‐window‐snp 1000 ‐‐homozyg‐window‐het 10 ‐‐homozyg‐window‐threshold 0.001 ‐‐homozyg‐kb 100. See Meyermans et al. (2020) for a description of these options and their effect on ROH identification.

### 2.6 Inferring selective sweeps from blockwise mutation configurations

We fit selective sweep models to 12 chromosome fusions by considering patterns of mutation within 1 Mb of sequence surrounding each rearrangement. Each fusion is private to one of the *Brenthis* species, that is, we did not include fusions shared by multiple species which likely fixed many generations ago. Two of the 12 fusions were not inferred by the maximum parsimony method described above. Syngraph inferred a single ancient fusion and then a subsequent fission in *B. daphne*. Independent fusions in *B. hecate* and *B. ino* are equally parsimonious and supported by the fact that different chromosome ends are involved in each case. We therefore include these potential fusions in our analysis.

We used the same species‐specific filtered VCF files described above as data for inferring selective sweeps. We annotated genes in the assemblies (see Supplementary Methods) and removed SNPs within exons (±10 bases), that is, we only consider variation within intronic or intergenic sequence. We chose to analyse *n* = 4 diploids for each species, selecting the set of individuals that minimized pairwise intraspecific *F*_st_. We summarized the sequence variation surrounding each fusion in terms of the blockwise site frequency spectrum (bSFS; Bunnefeld et al., 2015), setting a block size so that the average block contained 1.5 segregating sites. We used six_lineage_bSFS.py (see Data accessibility) to record the folded bSFS for six lineages by considering all possible sets of three diploids from *n* = 4. We then applied a *k*_max_ value of two using format_blocks.py (see Data accessibility), that is, we recorded exact mutation counts up to a value of 2 in each block and any count greater was summarized as >2. In summary, each block contains counts of folded singleton, doubleton and tripleton mutations from a sample of six genomes.

**FIGURE 1:**
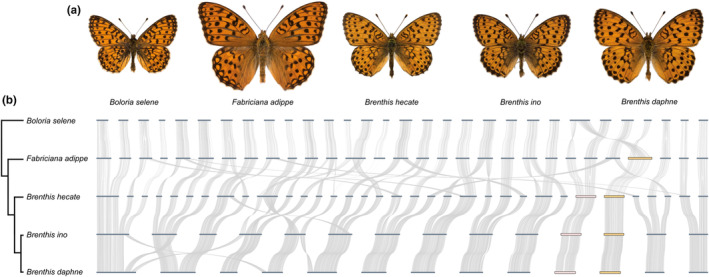
Synteny relationships between genomes of three *Brenthis* species, as well as species from two related genera. (a) Upper sides of male butterflies representing each of the five species. (b) A tree showing phylogenetic relationships between the species. The topology is from Chazot et al. (2021) and the plotted branch lengths are not to scale. Whole genome alignments are shown to the right of the tree. Thick horizontal bars are chromosomes and curved lines are nucleotide alignments, or, in the case of *B. selene* and *F. adippe*, shared BUSCO genes. Two sets of orthologous chromosomes are highlighted towards the right of the plot: an autosomal chromosome shared by all three *Brenthis* species (pink) and the Z chromosome that is shared by all *Brenthis* species and *F. adippe* (orange).

We implemented the sweep inference method of Bisschop et al. (2021) in *Mathematica* (see Data accessibility). In this method, the composite likelihood of a selective sweep is calculated by multiplying the probabilities of observing mutation configurations in short sequence blocks. The probabilities of different mutation configurations (bSFS entries) depend on the parameters of the sweep model (*θ*, *α* and *T*_a_, see Results) as well as the distance of a block from the sweep centre. For a given point in the genome, we estimated the composite likelihood of a neutral model and a selective sweep model given the bSFS counts in the surrounding 1 Mb region. We normalized the difference in composite likelihood (Δln CL) by the number of blocks to allow comparisons between 1 Mb regions with a different number of blocks. In cases where chromosome fusion points could only be narrowed down to intervals spanning >5 kb, we sampled points every 5 kb and reported parameter values for the point with the greatest Δln CL (Table 1). As a comparison, we also fit sweep models to points sampled from a non‐rearranged chromosome (Figure 1). Additional details of the model fitting procedure can be found in the Supplementary Methods.

**TABLE 1:** Maximum composite likelihood parameter estimates for selective sweeps around chromosome fusions in *Brenthis* butterflies.

Taxon	Chr	Position (mb)	Log_10_(*α*)	*T*_a_	Δln CL per‐block
*B. daphne*	1	36.7	−2.29	1.00	.000
*B. daphne*	1	45.2	−4.71	0.10	.013
***B. daphne***	**2**	**23.9**	**−5.67**	**0.08**	**.123**
*B. daphne*	3	7.4	−5.70	0.09	.086
*B. daphne*	8	6.7	−5.70	0.25	.016
*B. hecate*	2	6.8	−4.60	0.35	.007
*B. hecate*	2	19.4	−5.70	0.89	.012
*B. hecate*	5	15.0	−4.08	1.00	.000
*B. ino*	1	6.6	−4.43	1.00	.000
*B. ino*	3	24.1	−5.70	1.00	.008
*B. ino*	8	22.2	−5.70	1.00	.004
*B. ino*	9	7.4	−5.03	1.00	.000

*Note*: The sweep with the greatest statistical support is highlighted in bold.

### 2.7 Simulations

To quantify the power and accuracy of sweep inference based on the bSFS, we performed coalescent simulations with msprime v1.0.2 (Baumdicker et al., 2021) and applied the sweep inference scheme to the simulated data. Three different scenarios were simulated: a strong selective sweep (*s* = 0.005, *T*_a_ = 250,000 generations ago, with *N*_e_ = 500,000), neutral evolution (*N*_e_ = 500,000) and neutral evolution in a population with a similar demographic history to *B. daphne* (as inferred by fastsimcoal2). The mutation and recombination rates were set to *μ* = *r* = 2.9 × 10^−9^ per site per generation. Each simulation was replicated 100 times, where a single replicate consisted of a 1 Mb sequence sampled for *n* = 4 diploids.

### 2.8 Statistical analysis

We used resampling tests to evaluate whether chromosome fusions are enriched for selective sweeps when compared to loci elsewhere in the genome. We measured two statistics—the number of fusions with putative sweeps and the sum of Δln CL across all fusions in each species—and compared these with points sampled from a non‐rearranged chromosome (Figure 1). Resampling was species‐specific, that is, we sampled the same number of points as fusions analysed for each species. We generated 100,000 random sample sets and calculated one‐tailed *p*‐values as the proportion of samples with values greater than our observed statistics.

## 3 RESULTS

### 3.1 A genome assembly of *Brenthis hecate*

We generated a chromosome‐level genome assembly for *Brenthis hecate* using a combination of Pacbio long‐reads, Illumina short‐reads and HiC data (Figure S1). The assembly is 408.8 Mb in length, with a scaffold N50 of 12.8 Mb and a contig N50 of 5.9 Mb. Of the 45 sequences in the assembly, 34 are chromosome level (hereafter simply referred to as chromosomes), whereas the remaining 11 are contigs that could not be scaffolded (15–104 kb in length, totalling 409 kb). The chromosomes show a bimodal distribution in size (Figure S1), with seven large chromosomes (21.5–29.0 Mb) and 27 smaller chromosomes (6.6–14.0 Mb). The number of chromosomes in the *B. hecate* genome assembly (*n*_c_ = 34) is consistent with reports of spermatocytes sampled from both France and Siberia (de Lesse, 1961; Saitoh & Lukhtanov, 1988). The genome sizes of *B. hecate*, *B. daphne* and *B. ino* are all similar: 409, 419 and 412 Mb respectively.

### 3.2 Synteny between Argynnini butterfly species

To characterize chromosome rearrangements, we performed whole genome alignments between the three *Brenthis* species, as well as genome assemblies from two other fritillary butterfly genera in the tribe Argynnini. The whole genome alignments show that the two outgroup species, *Fabriciana adippe* and *Boloria selene*, have very similar genome/chromosome organization (Figure 1). By contrast, genomes of the *Brenthis* species show evidence for many rearrangements (Figure 1).

We next placed fission and fusion events on the phylogeny (*Brenthis* sp. and outgroups) using a maximum parsimony method (see Section 2). Of the 53 inferred rearrangements, 50 are found on branches leading to *Brenthis* species or their most recent common ancestors. The branch with the greatest number of inferred rearrangements (11 fissions and nine fusions) is that leading to the common ancestor of the three *Brenthis* species. Closer to the present, 14 fusion rearrangements are estimated on the branch ancestral to *B. daphne* and *B. ino*, while five fissions and two fusions are estimated on the branch leading to *B. hecate*. These rearrangements explain the large difference in chromosome number between these species. We also infer one fission and five fusions on the branch leading to *B. daphne* and three fusions on the branch leading to *B. ino*. Together, these rearrangements form a complex history of genome ‘reshuffling’ that is not seen in the outgroup lineages.

### 3.3 The demographic history of *Brenthis* butterflies

**FIGURE 2:**
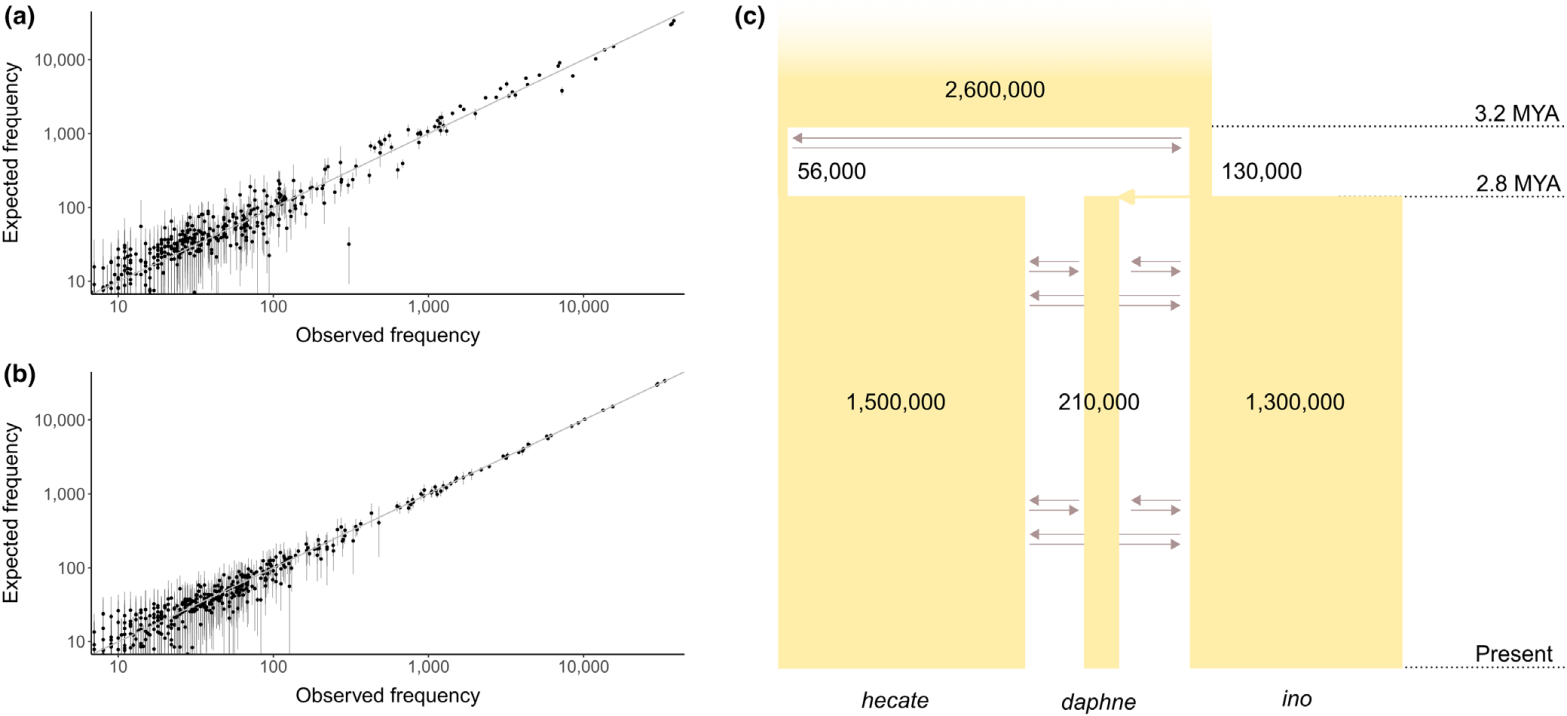
A demographic history of divergence and gene flow between three species of *Brenthis* butterfly. (a) A comparison between the expected and observed 3D‐SFS given the estimated demographic history. Each point represents a single SFS entry, with its position determined by its expected frequency (*y*‐axis) and the frequency observed in the data (*x*‐axis). The diagonal line (*x* = *y*) represents a perfect fit between the model and the data and errors bars represent 95% CIs estimated from simulation replicates. (b) The same as in (a) but observed frequencies are derived from a single simulation. The correlation therefore represents the expected fit when data are simulated under that exact model. (c) A schematic representing the estimated demographic history. Each rectangle represents a population with width proportional to effective size (*N*_e_). The *N*_e_ of each population is also given to two significant figures. Grey horizontal arrows represent the fact that contemporaneous populations exchange migrants in both directions. The timing of speciation events is shown on the right of the plot in units of a million years (1 generation = 1 year).

To estimate the timing of rearrangements as well the effective size of the populations in which they fixed, we inferred a multispecies demographic history using allele frequencies in resequenced genomes (see Section 2). The best‐fitting demographic model estimates the *B. daphne* and *B. ino* split at 2.8 MYA and the split with *B. hecate* at 3.2 MYA (Figure 2c). These speciation times allow for an estimation of the rearrangement substitution rate per genome and generation: 3.3 × 10^−6^, that is, one rearrangement every ~300 k generations.

Overall genetic diversity in these species (~1% at 4D sites, Table S3) is typical of butterflies (Ebdon et al., 2021; Mackintosh et al., 2019), suggesting that long‐term *N*_e_ is on the order of 10^5^ or greater. We co‐estimated *N*_e_ and *m*_e_ parameters within the multispecies demographic model, thus taking into account the effect of interspecific gene flow on diversity. We find that *N*_e_ estimates of species and ancestral populations vary but are generally high, as expected (Figure 2c). The population in which the most rearrangements fixed (the ancestor of *B. daphne* and *B. ino*) has a relatively small *N*_e_ (1.3 × 10^5^). By contrast, the population in which the fewest rearrangements fixed (*B. ino*) has a much larger *N*_e_ (1.3 × 10^6^). While this may hint at a negative relationship between *N*_e_ and rearrangement rate, we cannot meaningfully test this from such a small species tree. Nonetheless, the fact that these species have large effective population sizes, as is typical of insects, suggests that rearrangements do not require extremely small long‐term *N*_e_ to become fixed. However, this demographic model only partially fits the data (Figure 2a,b). The parameter estimates for ancestral populations also have wide 95% CIs (Table S2), and therefore, the *N*_e_ estimates from this model are only approximate (see Section 4).

### 3.4 Runs of homozygosity

**FIGURE 3:**
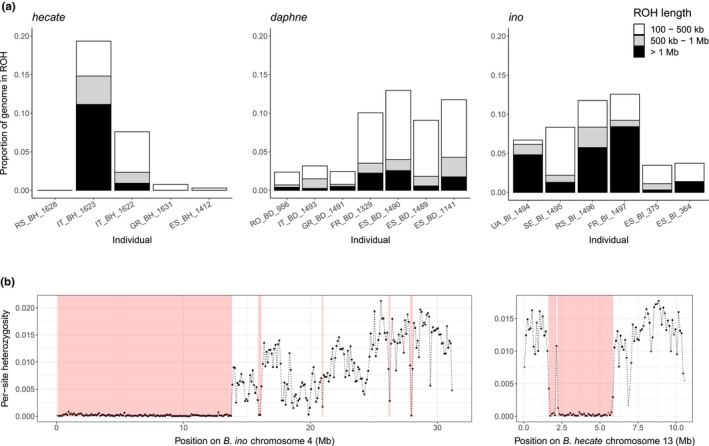
Evidence for inbreeding among *Brenthis* butterflies. (a) The fraction of the genome covered by runs of homozygosity (ROH) in each *Brenthis* individual. (b) Per‐site heterozygosity for *B. ino* individual FR_BI_1497 plotted in 100 kb windows across chromosome 4, and the same for *B. hecate* individual IT_BH_1623 across chromosome 13. Red shading shows regions that were identified as ROH.

The SFS contains information about long‐term *N*_e_, whereas regions of the genome that are identical by descent are informative about *N*_e_ in the recent past. With this in mind, we searched for runs of homozygosity (ROH) within individual genomes. Large ROH (>=1 Mb) are generated through recent shared ancestry and should be rare (for *N*_e_ ≈ 10^5^) or almost absent (for *N*_e_ ≈ 10^6^) in well‐mixed populations. For example, the probability that a 1 Mb window is covered by a ROH in a population with effective size equivalent to *B. ino* is 7 × 10^−5^ when assuming a conservatively low recombination rate of *r* = 2.9 × 10^−9^. This corresponds to a probability of only 0.0284 that at least one ROH >=1 Mb is observed within a 412 Mb genome. Surprisingly, we found that the majority of individuals across all three species (15 of 18) have at least one ROH of this size (Figure 3a). Summing the length of these ROH to estimate the inbreeding coefficient *F*_roh_ reveals that there are several individuals with *F*_roh_ ≈ 1/16 (Figure 3), consistent with being the offspring of first cousins. These results suggest that short‐term *N*_e_ within local populations may be much lower than indicated by overall, that is, species‐wide, levels of diversity or predicted by the SFS‐based model of demographic history. Although smaller local populations may promote the fixation of rearrangements through drift, it is less clear whether this would lead to fixation across the entire species range (see Section 4).

### 3.5 Parameter estimates and statistical support for simulated selective sweeps

**FIGURE 4:**
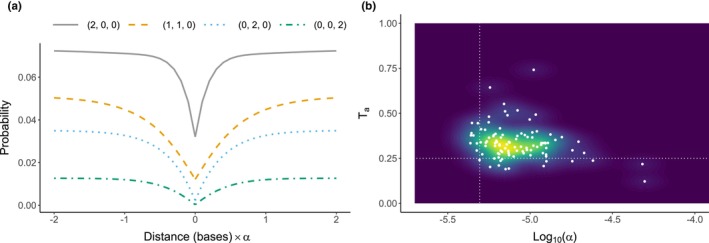
Inferring sweeps from the bSFS. (a) The probability of observing particular bSFS entries (*y*‐axis) given the distance of a block from the sweep centre (*x*‐axis). In this example, the sweep occurred 0.5*N*_e_ generations ago (i.e. *T*_a_ = 0.25) and the population mutation rate is 0.66 (e.g. *θ*_per‐site_ = 0.0058 and block length = 113 bases). For a sample of six lineages with folded mutations and counting up to two mutations per branch type, there are 64 total bSFS entries. Each line represents one of these entries, where (*i*, *j*, *k*) denotes a block with *i* singleton mutations, *j* doubletons and *k* tripletons. The x‐axis shows that the effect of a sweep at a particular locus depends on the relative strength of the sweep (*α*) and the distance of that locus from the sweep centre. (b) Parameter estimates for 100 simulation replicates of a selective sweep. The true sweep strength (Log_10_ (*α*), *x*‐axis) and timing (*T*_a_, *y*‐axis) are shown with dotted vertical and horizontal lines respectively. Each point represents parameter estimates for a single simulation replicate, and coloured contours show the density of these estimates across all replicates.

It is possible that the rearrangements observed in this genus have become fixed through natural selection rather than drift (see Introduction). To test this, we ask whether loci surrounding chromosome fusions show evidence for selective sweeps. The sweep inference presented in Bisschop et al. (2021) calculates the likelihood of a hard selective sweep given mutation counts in short sequence blocks (the bSFS). While Bisschop et al. (2021) used unfolded mutation counts for four lineages, this requires polarization, that is, knowledge of ancestral states. We can only obtain this information for genic regions of the genome given the considerable divergence between *Brenthis* sp. and the nearest available outgroup, *F. adippe* (~0.09 at 4D sites). We therefore adapted the composite likelihood‐based sweep inference to folded mutation counts for six lineages (Figure 4).

We first tested whether this implementation can accurately infer old sweeps. We simulated strong selection (*N*_e_ *s* = 2500, see Section 2) and estimated the statistical support for a sweep while also obtaining maximum composite likelihood estimates (MCLE) for three parameters: *θ*, *α* and *T*_a_. Here, *θ* = 4*N*_e_*μ*l is the population mutation rate per block (where *l* is the block length), *α* = *r*/*s* ln[2*N*_e_*s] is the rate of recombination relative to the strength of the sweep and *T*_a_ is the timing of the sweep in units of 2*N*_e_ generations. Across simulations, we find that the statistical support for a sweep—that is, the increase in composite likelihood (Δln CL) compared to the best‐fitting neutral model—is always non‐zero with a median Δln CL per block of 0.018. The per block mutation rate (*θ* = 0.66 for l = 113) is well estimated, albeit with a small downward bias (lower quartile, median, upper quartile = 0.62, 0.64, 0.65 respectively). Similarly, the timing (*T*_a_) and strength (*α*) of the sweep are slightly overestimated and underestimated respectively (Figure 4). These results show that sweep parameters can be inferred through this method under a simple demographic history.

Repeating this analysis but simulating entirely neutral evolution (see Section 2) leads to inferred sweeps that are very weak or, in a minority of cases, strong but very old (Figure S2) and weakly supported: the median Δln CL across these simulations is 3.3 × 10^−5^ and the maximum is 0.002. Given these results, we use thresholds of Log_10_(*α*) < −4 and Δln CL > 0.002 to define plausible sweep candidates. This *α* value implies a distortion of genealogical branch lengths across at least 20 kb (Figure 4) and corresponds to *s* = 1.7 × 10^−4^, given an *N*_e_ of 1 × 10^6^ and a recombination rate of 2.9 × 10^−9^. At our chosen thresholds, we may discard some weak selective sweeps but false positives should be rare.

### 3.6 Evidence for an enrichment of selective sweeps around chromosome fusions

We next applied the same inference procedure to a total of 12 potentially recent chromosome fusions, with five, three and four fusions sampled from *B. daphne*, *B. hecate* and *B. ino* respectively. Four fusions show no statistical support for a selective sweep (Table 1). The remaining eight fusions have Log_10_(*α*) and Δln CL values that our simulations suggest are unlikely to be observed under neutral evolution (see above). To test whether chromosome fusions have greater statistical support for selective sweeps than other regions of the genome, we fit sweep models across an entire chromosome for each species (~250 points spaced 100 kb apart). We chose the same orthologous chromosome for each species—the only autosome that has not undergone any rearrangements within the genus (Figure 1). Summing the Δln CL of the 12 fusions and comparing this to points sampled from these non‐rearranged chromosomes suggests that there is no strong enrichment for signals of selective sweeps (observed = 0.269, one‐tailed 95% CIs of permutations = [0, 0.349], one‐tailed *p*‐value = .161). Similarly, although eight of the 12 fusions show evidence of a selective sweep, this result is not a significant departure from what can be obtained by sampling points from the non‐rearranged chromosomes (one‐tailed 95% CIs of permutations = [0, 8], one‐tailed *p*‐value = .060).

The fact that we infer sweeps around some chromosome fusions, but that this is unremarkable when compared to other regions of the genome, suggests a much higher false‐positive rate in the real data than in our idealized neutral simulation check. Considering points sampled across the non‐rearranged chromosome, we find that 26.9% are classified as sweeps both in *B. hecate* and *B. ino*, although the vast majority of these are old (*T*_a_ > 0.5, Figure S2). In *B. daphne*, the frequency of inferred sweeps is even higher at 63.2%, and, in contrast to the other species, these sweeps are almost always estimated to be recent (*T*_a_ ≈ 0.1, Figure S2). A plausible explanation for this is that gene flow into *B. daphne* from *B. ino* has generated genealogical histories that are better explained by a model of recent sweeps than a single panmictic population. Simulating data for a single population that has undergone the long‐term demographic history inferred for *B. daphne* (Figure 2c) and fitting a sweep model to these data, we recovered false‐positive sweeps (32.0% of simulations), albeit with older inferred ages (*T*_a_ ≈ 0.5, Figure S2). This suggests that gene flow and changes in *N*_e_ over time likely explain at least some of the signatures of selective sweeps around chromosome fusions.

### 3.7 Evidence for individual selective sweeps

**FIGURE 5:**
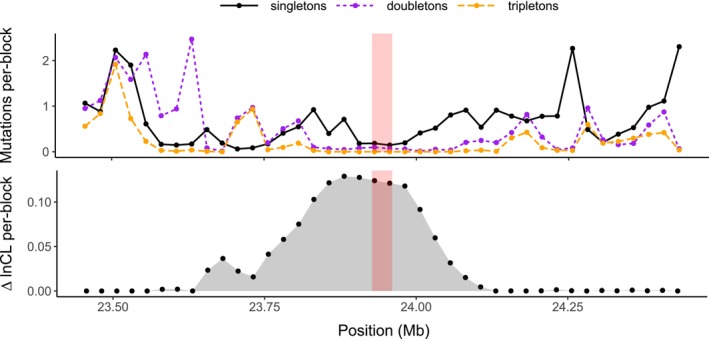
A potential selective sweep around a chromosome fusion on *B. daphne* chromosome 2. The top panel shows the frequency (*y*‐axis) of the three folded mutation classes across the 1 Mb of sequence that surrounds the fusion point (*x*‐axis). Mutation frequencies are plotted in 25 kb windows. The bottom panel shows the statistical support for a selective sweep (*y*‐axis) across the same region. Support is measured as the difference in composite likelihood (Δln CL) between a selective sweep model and a neutral model. The models were fit at 40 test points at 25 kb intervals across this region, each represented by a point in the plot. The transparent red bar in the centre of both panels marks a 33 kb region which contains the fusion point.

We next consider the strength of evidence for individual sweeps around chromosome fusions. The fusion with the strongest sweep support is on chromosome 2 of the *B. daphne* genome and has a per‐block Δln CL (Figure 5) that is greater than 95% of points sampled from the non‐rearranged chromosome. The inferred sweep parameters (*θ* = 0.85 for *l* = 210, Log_10_(*α*) = −5.7, *T*_a_ = 0.079) correspond to an *s* of 0.0012 and a timing of 56 k generations ago when assuming *μ* = *r* = 2.9 × 10^−9^. Visualizing counts of folded mutation classes shows a scarcity of tripleton and doubleton mutations near the fusion, as well as an overall reduction in diversity (Figure 5). In fact, there is a 264 kb region which encompasses the fusion point that does not have a single tripleton mutation (i.e. a mutation shared by three out of six lineages, Figure 5).

It is possible that the reduction in diversity around this chromosome fusion has been generated by processes other than a selective sweep, for example, a lower de novo mutation rate or background selection. We therefore fit an alternative model to this region in which the fusion point is encompassed by a local reduction in *θ* that extends for *d* bases in either direction. We find that this model of locally reduced diversity (*θ* = 1.17, *θ*_local_ = 0.40, *d* = 400 kb) fits better than the neutral model with a single parameter (*θ* = 0.55, per‐block Δln CL = 0.110) but not as well as the sweep model (per‐block Δln CL = −0.013). Finally, we also test whether the sweep is supported when considering all seven *B. daphne* genomes, rather than just the four originally analysed. Under this sampling, the inferred sweep is of a similar strength but older (*T*_a_ = 0.26) and with reduced statistical support (per‐block Δln CL = 0.057 rather than 0.123). Given the confounding effects of demography, we must interpret patterns of mutation around this chromosome fusion carefully. Nonetheless, our results do raise the possibility that this chromosome fusion has risen in frequency due to positive natural selection.

## 4 DISCUSSION

### 4.1 Patterns of chromosome evolution

Chromosome rearrangements are a fundamental part of eukaryote genome evolution, yet some groups of organisms display a much higher rate of rearrangement than others. We have focused on one such taxon, butterflies in the genus *Brenthis*, and have shown that these species have undergone a complex history of chromosome rearrangement not shared by other closely related genera (Figure 1). We find evidence for a large number of chromosome fusions as well as several fissions, with multiple rearrangements occurring between speciation events. We have assumed that chromosomes rearrange through fissions and fusions, rather than translocations. Although small segments of chromosomes appear to have translocated between *B. daphne* and *B. ino* chromosomes (Figure 1), the fact that these segments are single chromosomes in *B. hecate* suggests that these are ancestral chromosomes that have fused differentially. Overall, the pattern and tempo of rearrangement appears similar to what has been described in *Melinaea* butterflies (Nymphalidae; Gauthier et al., 2022) and dissimilar from genera such as *Lysandra* (Lycaenidae) that are dominated by chromosome fissions (Wright et al., 2023). While it is perhaps unsurprising that the mode of rearrangement evolution differs between lineages, there do appear to be some shared features. For example, the Z sex chromosome is one of just two chromosomes that are not rearranged between *B. hecate* and *B. daphne*, and although we have previously identified a Z‐autosome fusion in one *B. ino* haplotype (Mackintosh et al., 2022), this rearrangement is not fixed. The Z is also the only chromosome that has not undergone extensive fissions in *Lysandra* sp., and it is one of only two chromosomes that are not rearranged between *Melinaea marsaeus* and *M. menophilus* (Gauthier et al., 2022). It therefore seems likely that rearrangements involving the Z chromosome have different fitness effects than autosomal rearrangements (Wright et al., 2023).

### 4.2 Genetic drift and underdominance

Fissions and fusions are likely to be underdominant, that is, deleterious when in a heterozygous state, because proper pairing and segregation of chromosomes during meiosis is often impaired (Grize et al., 2019; Nunes et al., 2011, although see Borodin et al., 2019; Mercer et al., 1992). In that case, fixation of these rearrangements is due to strong genetic drift in small populations (Wright, 1941). To investigate this possibility in *Brenthis* butterflies, we face a conundrum: The chromosome rearrangements we have investigated likely fixed at different time points spread across millions of years (Figures 1 and 2) for which we only have information about the long‐term coalescent *N*_e_. However, it is the short‐term *N*_e_ that determines the fixation probability of a new mutation, and our observation of considerable ROH (Figure 3) suggests that this may be much lower than our long‐term estimates. We therefore explore the fixation probability of a new rearrangement in both contexts.

Our estimates of long‐term *N*_e_ from the SFS are on the order of ~10^5^, with some variation between species and over time (Figure 2c). Given that we have estimated *N*_e_ ~ 10^5^ and the rate of rearrangement fixation as 3.3 × 10^−6^, we can use the fixation rate of Lande (1979) to estimate an upper bound on the heterozygote disadvantage of rearrangements. Although we do not know the de novo rearrangement rate, we can assume that it is no higher than one rearrangement per genome per generation, as otherwise most individuals would be heterozygous for multiple new fissions or fusions (which we do not observe in our genome assemblies). Under this very conservative assumption, the maximum heterozygote disadvantage is *s* = 1.4 × 10^−4^, suggesting that heterozygosity for a fission or fusion has a weak absolute fitness effect in these species.

The above calculation assumes a large panmictic population, which is at odds with our observation of ROH within individual genomes (Figure 3). Observations of large long‐term *N*_e_ yet considerable ROH can be reconciled by considering population structure within species. As an illustration, we consider the simplest possible scenario—a finite‐island model (Maruyama, 1970)—with ROH providing information about the proportion of recent within‐deme coalescence. By fitting this model to three summary statistics (*H*, *d*_xy_ and *W*_roh_, see Supplementary Methods), we find that a meta‐population with 260 demes, each with an *N*_e_ of 3400 and an *m*_e_ of 4 × 10^−4^, has the same expected levels of diversity, divergence and ROH as found among *B. ino* individuals. We stress that this calculation assumes the simplest possible model which is unlikely to capture the complex population structure that exists within these dispersive species. Nonetheless, it suggests that local populations of *Brenthis* butterflies may have a short‐term *N*_e_ that is at least an order of magnitude smaller than overall diversity would suggest.

Is it then possible that population structure has facilitated the fixation of deleterious rearrangements through genetic drift? In the absence of migration, we can perform the same calculation as above for a local population with *N*_e_ = 3400, and we find a much higher upper bound of *s* = 0.004. Fixation in the total population, however, requires low levels of migration so that the rearrangement can still establish locally and spread through the population by extinction and re‐colonization events (Lande, 1979; Spirito et al., 1993). The *m*_e_ values we infer under a finite‐island model suggest that migration between demes is high (4*N*_e_ *m*_e_ > 1, Table S3), in which case population structure can only have a weak effect on the fixation probability of an underdominant rearrangement (Slatkin, 1981). We therefore conclude that there is not enough population structure in these butterfly species—at least in the very recent past—to allow the fixation of strongly underdominant rearrangements. Furthermore, given that we ignore the effect of selection at linked sites (Corbett‐Detig et al., 2015; Maynard Smith & Haigh, 1974), we are likely underestimating the short‐term *N*_e_ of these butterfly species and therefore overestimating the probability that any of the chromosome rearrangements that have fixed in these species have appreciable underdominant fitness effects.

The idea that rearrangements in these species have only very small deleterious fitness effects is further supported by the fact that *B. daphne* and *B. ino* can produce fertile hybrids (Kitahara, 2008, 2012) despite their karyotypes differing by as many as nine rearrangements (Figure 1). It is not clear how meiosis in these species is so robust to the risk of improper segregation in the presence of heterokaryotypes, although inverted meiosis is one potential explanation that has been described for other butterflies (Lukhtanov et al., 2018, 2020). While we cannot calculate the exact fitness effects of rearrangements in *Brenthis* butterflies, we can at least rule out the possibility that strongly underdominant rearrangements (e.g. *s* > 0.01) have fixed through genetic drift.

### 4.3 The role of positive natural selection in the fixation of chromosome fusions

The scenario in which fusions are favoured by natural selection would mean that they play a role in adaptation (Guerrero & Kirkpatrick, 2014; Yeaman, 2013) and/or that they are driving as selfish elements. There is currently little empirical evidence that fusions fix through positive natural selection (although see Stewart et al., 2019), but this is unsurprising given that the majority of identified fusions are relatively old. For example, chromosome 2 of the human genome is the product of a fusion that happened approximately 900 kya (Poszewiecka et al., 2022), corresponding to ~3.6*N*_e_ generations in the past. Inferring the evolutionary history of such old mutations is challenging given the fact that, on average, all but two lineages in a genealogical tree coalesce within 2*N*_e_ generations. We have therefore focused on species with recent chromosome fusions and a large long‐term *N*_e_ (Figure 2c), giving us some power to detect the effects of natural selection.

We fit selective sweep models to 12 chromosome fusions and found that the aggregate statistical support is greater than what is found when sampling from a non‐rearranged chromosome, but not significantly so. The simplest explanation for this result is that these fusions are selectively neutral and fixed by genetic drift. However, we cannot rule out of the possibility that at least some fusions fixed through positive selection but did so >2*N*_e_ generations ago, with only a subtle signal remaining in present‐day genome sequence data (Bisschop et al., 2021). We nonetheless interpret this result as evidence against a scenario where the majority of chromosome fusions fix through very strong selection, such as (holocentric) meiotic drive.

One fusion in our data set, that on *B. daphne* chromosome 2 (Figure 5), has greater statistical support for a sweep than 95% of points sampled elsewhere in the genome. However, since we have considered 12 fusions, the probability that at least one meets this threshold by chance is considerable (*p* = .46). This fusion does, however, have greater support for a sweep than all 100 simulations performed under the *B. daphne* demographic history inferred under a multispecies model, and so sequence variation in this region cannot easily be explained by demography alone. The reduction in diversity around this chromosome fusion (Figure 5) could be explained by a recombination desert in which background selection continuously erodes diversity. Although this ad hoc explanation could be applied to almost any inferred sweep, it is at least plausible in this case as the fusion point is in the centre of the chromosome where recombination is typically lowest in butterfly genomes (Palah i Torres et al., 2022; Shipilina et al., 2022). Additionally, the fact that these fusions act as barriers to gene flow (Mackintosh et al., 2023) is another explanation for the reduction in diversity that we observe. A more general issue is that selective sweep signatures can also be generated by the fixation of deleterious mutations (Johri et al., 2021). This is because mutations with fitness effects *s* and −*s* have the same expected fixation time (Maruyama & Kimura, 1974). Such mutations would, however, have very different fixation probabilities so long as 2*N*_e_ *s* >> 1. We estimate 2*N*_e_ *s* ≈ 850 for the sweep on *B. daphne* chromosome 2, making the fixation of a deleterious chromosome fusion an unlikely explanation for the sweep signature in this region.

Some uncertainty remains as to whether the inferred selective sweep around the chromosome fusion on *B. daphne* chromosome 2 is a true positive, and we therefore interpret our results as weak evidence for the idea that chromosome fusions primarily fix through positive natural selection. The patterns of mutation around this particular fusion are nonetheless unusual and so warrant further exploration. Ideally, future analyses will jointly model the effects of demography and natural selection on sequence data, which is a long‐standing goal in population genomic inference (Jensen et al., 2005; Lauterbur et al., 2022; Przeworski, 2002).

### 4.4 Outlook

Knowledge about how genomes change over time is key for our understanding of evolution. Although fission and fusion rearrangements represent just a small fraction of the ways in which genomes can change, we know particularly little about how these drastic mutations become fixed in populations. To address this, we have analysed genome‐wide variation in *Brenthis* butterflies to infer past demography and natural selection in relation to chromosome rearrangements. Our main findings are that (i) drift is not strong enough to fix considerably underdominant rearrangements and (ii) there is only weak evidence that chromosome fusions fixed through positive natural selection or meiotic drive. We cannot yet construct a full model of how rearrangements fix in these species, but our results are consistent with rearrangements having small fitness effects and fixing through drift. Clearly, other types of information not contained in genome sequence data are required for a full picture of how rearrangements fix. For example, direct estimates of heterokaryotype fitness (Knief et al., 2016; Luo et al., 2018) and de novo rates of rearrangement (Yamaguchi & Mukai, 1974) are invaluable for understanding rearrangement evolution. Additionally, while we have focused on rearrangements that are likely to have fixed recently, an alternative strategy would be to identify and analyse the small subset of rearrangements that are still segregating within a species. It is more challenging to collect data on such examples, but they could provide information about how rearrangements rise (and fall) in frequency over time. The population genomic analyses presented here represent a first step in understanding how fission and fusion rearrangements fix in *Brenthis* butterflies. We anticipate and look forward to similar investigations in other groups of organisms where chromosome rearrangements are common, which together will illuminate how genomes evolve across the tree of life.

## AUTHOR CONTRIBUTIONS

AM, SHM, DS and KL designed the research. AM and DS wrote code for analysis. AM analysed genomic data. AM wrote the manuscript with input from RV, SHM, DS and KL.

## CONFLICT OF INTEREST STATEMENT

The authors declare no conflict of interest.

## Supporting information


Data S1

## Data Availability

All new sequence data generated in this study and the *Brenthis hecate* genome assembly are available at the European Nucleotide Archive under project accession PRJEB62818. Data associated with the population genomic analyses are available at Dryad: https://doi.org/10.5061/dryad.cnp5hqcbf. Python scripts and *Mathematica* notebooks are available at the following Github repository: https://github.com/A‐J‐F‐Mackintosh/Mackintosh_et_al_2023_rearrangement_fixation.
