# Supplementary material for: Do chromosome rearrangements fix by genetic drift or natural selection? Insights from Brenthis butterflies
Source: Mol Ecol. 2023 Oct 9;33(24):e17146. doi: 10.1111/mec.17146 (PMC11628658; doi:10.1111/mec.17146)
Supplement: Supplementary file 1 — Data S1 [file MEC-33-e17146-s001.pdf]

Supplementary Materials

Supplementary Methods

Gene annotation

We annotated genes in three genome assemblies: *F. adippe*, *B. daphne*, and *B. hecate* (note that *B. ino* already has a gene annotation from Mackintosh *et al.* 2022). This was done so that the SFS-based demographic modelling could be restricted to putatively neutral fourfold-degenerate (4D) sites in the *F. adippe* genome, and that exonic regions in the *Brenthis* genomes could be excluded when fitting sweep models. We masked repeats in the *B. daphne* and *B. hecate* genomes using Red (Girgis 2015) with default parameters. A repeat-masked version of the *F. adippe* assembly was kindly supplied by Tobias Baril (personal communication), having been repeat annotated with EarlGrey v1.2 (Baril *et al.* 2021, 2022).

RNA-seq data was generated for an *F. adippe* and *B. hecate* individual and kindly shared with us by Sam Ebdon (Table S1). RNA extractions, library preparations, and sequencing were performed alongside datasets generated for Ebdon *et al.* (2021). We also accessed the RNA-seq dataset for *B. daphne* from Ebdon *et al.* (2021). We next mapped species-specific RNA-seq reads to the assemblies with HISAT2 v2.1.0 (Kim *et al.* 2019). The repeat-masked assemblies and RNA-seq alignments were used as input for gene annotation with braker2.1.5 (Stanke *et al.* 2006, 2008; Li *et al.* 2009; Barnett *et al.* 2011; Lomsadze *et al.* 2014; Buchfink *et al.* 2015; Hoff *et al.* 2015, 2019). We used GenomeTools v1.6.1 (Gremme *et al.* 2013) to format gff3 and bed files for each annotation. Finally, 4D sites in the *F. adippe* genome assembly were identified with partition_cds.py (see Data accessibility).

Fitting a multi-species demographic model with fastsimcoal2

We fit a single demographic model to the folded 3D_SFS using fastsimcoal2 (version fsc27093). We chose to fit a complex model (Main text Figure 2) and then quantify the uncertainty in parameter estimates through parametric bootstraps (Table S2). To obtain maximum composite likelihood estimates for each parameter we performed the following optimisation command ten times:

```
fsc27093 -t brenthis.tpl -n 1000000 -m -e brenthis.est -M -L 30 -c 50 -B 50
```

This corresponds to parameter optimisation where each likelihood estimate is approximated using 1,000,000 coalescent simulations and parameters are optimised through Brent's algorithm across 30 rounds.

Given the parameter estimates with the greatest composite likelihood (Table S2), we performed 100 parametric bootstrap simulations with the following command:

`fsc27093 -i brenthis.par -n100 -c 5 -B 5 -j -m -s0 -x -I -q --multiSFS`.

Each simulation consists of 619 loci of length 4 kb with mutation and recombination rates of $\mu = r = 2.9 \times 10^{-9}$. This number and length of loci corresponds to the total amount of data used to generate the observed SFS, as well as the approximate level of linkage given that reads only map to genic regions of the *F. adippe* genome. Parameter estimates were then obtained for each simulated SFS using the same optimisation procedure as described above. These estimates were used to estimate 95% CIs (Table S2).

Strategies for fitting sweep models to the bSFS

We fit hard selective sweep models using the method of Bisschop *et al.* (2021). For each analysis we used data from 1 Mb of sequence and therefore thousands of short sequence blocks. Each composite likelihood calculation requires the probability of observing the mutation configuration (bSFS entry) of each block given its distance from the sweep centre. Instead of performing these calculations repeatedly, which would be prohibitively slow, we generated a grid with dimensions corresponding to θ, $\alpha * distance$ and T_a, in which, each element contains the exact probabilities of all 64 possible bSFS entries. The probability of a bSFS entry for a particular parameter combination and distance from the sweep centre can then be obtained through linear interpolation between points in the grid. The grid contained 15 θ points between 0.1 and 1.5, 47 $\alpha * distance$ points between 0 and 12.0, 11 T_a points between 0 and 1.0, and therefore 7755 parameter combinations in total. This places a limit on the age of sweeps that can be inferred ($T_a = 1$, i.e. $2N_e$ generations ago). When a sweep is weak, many blocks will be $\alpha * distance > 12$ away from the sweep centre and therefore outside of the grid. However, probabilities at such a high $\alpha * distance$ are effectively the same as under a neutral model, and we approximate the probability as such.

For a given point in the genome and the blockwise data in the surrounding 1 Mb, we optimised the parameters of the sweep model using the Nelder Mead algorithm in *Mathematica*. We repeated

the optimisation three times with different random seeds and retained the parameters with the greatest likelihood. We set a minimum $Log_{10}(\alpha)$ value of -5.7. This corresponds to a very strong sweep where $\alpha \times 500$ kb $= 1$. Sweeps with smaller α values than this would be unlikely to show a spatial pattern across 1 Mb and so cannot be identified reliably.

We fit two other models to the same data. The first is a neutral model with a single parameter, θ. The second is a model with a central region $2 * d$ bases in size where θ is reduced relative to a background value. Unlike the sweep model, these models do not include any distortion in genealogical branch lengths. Code for fitting all three of these models to bSFS data can be found in the *Mathematica* notebook titled brenthis_sweeps_chromosome_scan.nb (see Data accessibility).

Fitting a finite-island model

We tested whether a finite-island model (Maruyama 1970) could explain levels of overall genetic diversity and ROH in each *Brenthis* species. This model consists of local populations (i.e. demes) of effective size N_e, where the effective migration rate m_e is the per-generation probability that a lineage migrates out of a deme. The N_e of each deme largely determines the chance of very recent common ancestry. By contrast, the longer term rate of coalescence and therefore the overall levels of genetic diversity and divergence are a function of the effective migration rate (m_e) and number of demes.

We fit this finite-island model using three summary statistics: per-site heterozygosity (H), pairwise intraspecific divergence (d_{xy}) between individuals sampled from different demes in Europe, and the proportion of 1 Mb windows covered by a ROH (which we call W_{roh}). These statistics provide information about the rate of coalescence within and between demes (H and d_{xy}) as well as the rate of very recent within-deme coalescence (W_{roh}). For a given species, we averaged these statistics across all individuals/pairwise comparisons. We used the expected time of coalescence for lineages sampled within and between demes (Nagylaki 1982; Strobeck 1987; Wakeley 1999) to calculate the expected H and d_{xy}, respectively. We estimated the probability of observing a 1 Mb window covered by a ROH as the probability that, for two lineages sampled from the same deme, the first event backwards in time is coalescence rather than migration or recombination within the window. These calculations assume equal recombination and mutation rates ($\mu = r = 2.9 \times 10^{-9}$). We then inferred the parameters of the finite-island model (number of demes, N_e and m_e) as

those for which the expected H, d_{xy} and W_{roh} match the data. Model fitting was performed in a *Mathematica* notebook (finite_island_model.nb, see Data accessibility).

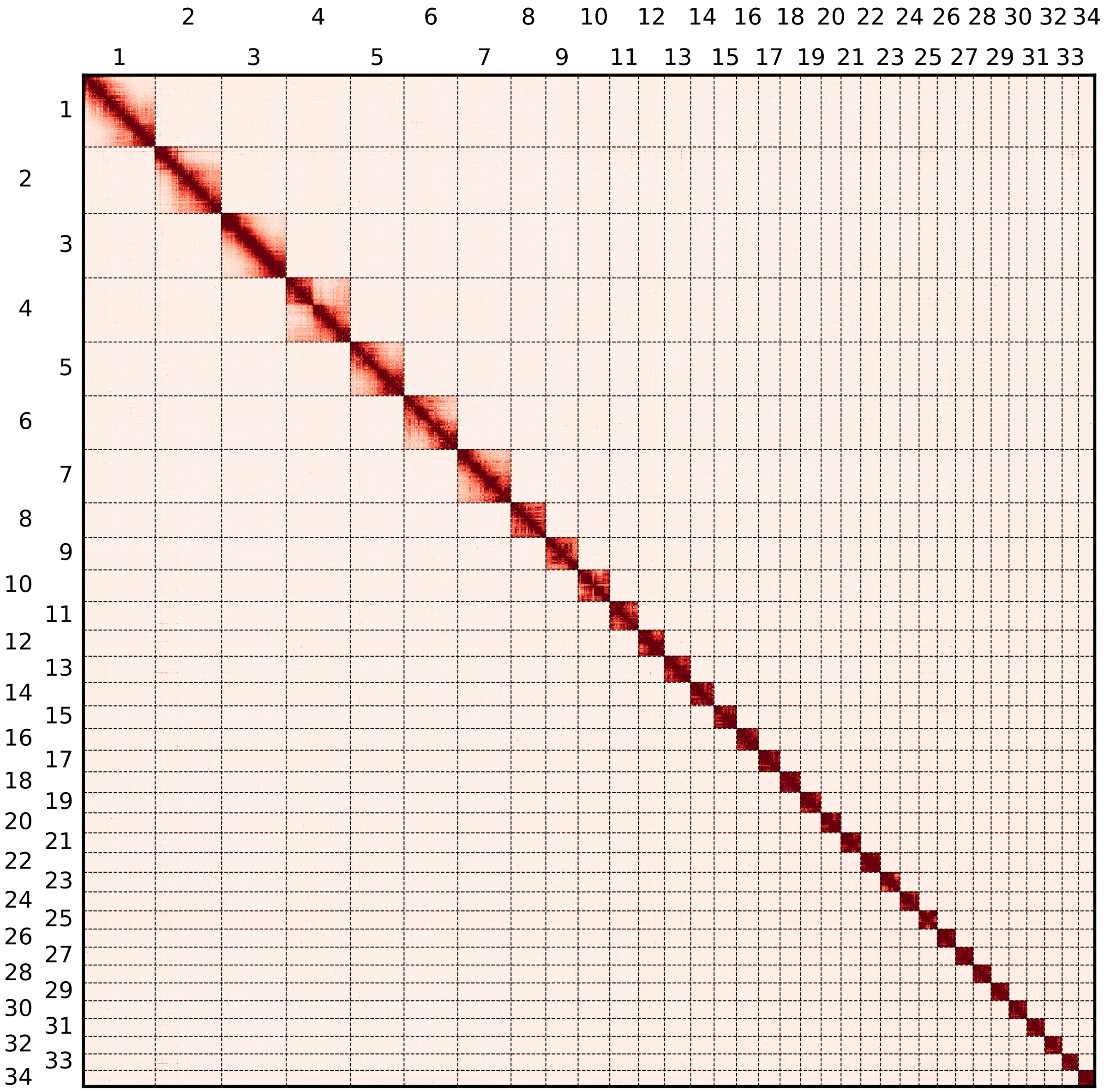

Figure S1: A HiC contact heatmap showing the 34 *Brenthis hecate* chromosomes.

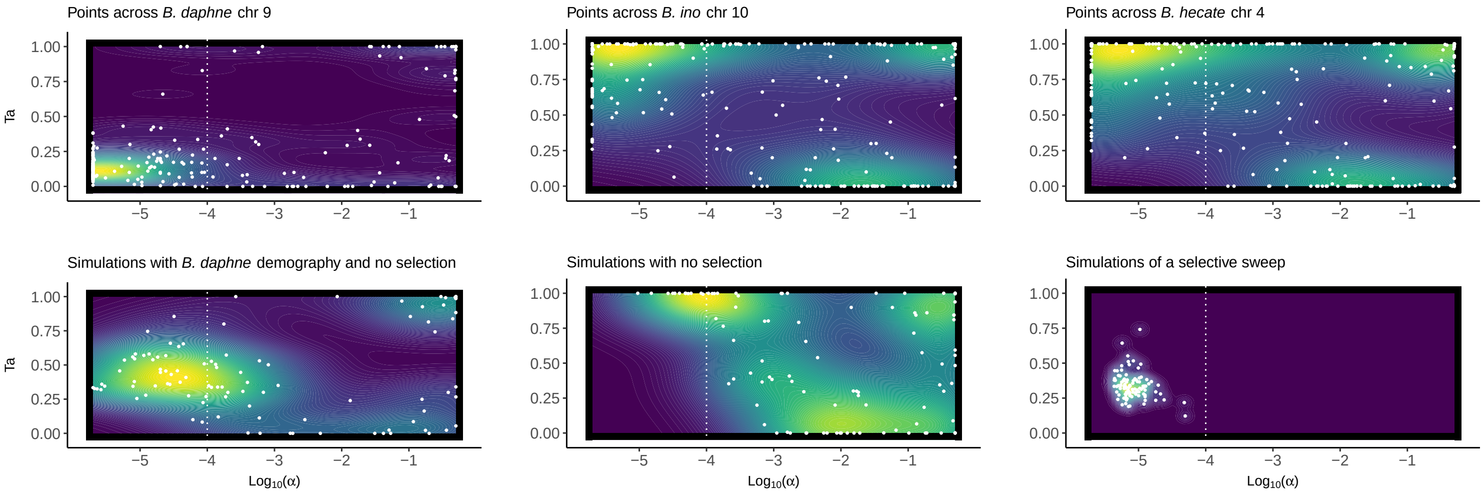

Figure S2: Parameters of inferred selective sweeps. Plots show the estimated strength of sweeps ($Log_{10}(\alpha)$, x-axis) and their estimated timing (T_a, y-axis). Within a plot, each white point represents parameter estimates for a test site in the genome or a single simulation, whereas coloured contours show the density of these estimates across multiple points/simulations. The top plots show inferred sweep parameters for points sampled across the same (orthologous) chromosome, for *B. daphne*, *B. ino*, and *B. hecate*. The bottom plots show inferred sweep parameters for simulations. Each plot has a vertical dashed line at $Log_{10}(\alpha) =$ -4, as points to the left of this can be considered putative selective sweeps (see Main Text).

Table S1: Sampling locations and other metadata for the individuals used to generate new sequence data in this study.

Sample	Preservation	Date	Species	Sex	Locality	Region	Country	Lat	Long	Collector	Data
ES_BH_1411	Liquid nitrogen	6/6/2019	Brenthis hecate	Male	Segura de la Sierra, Jaén	Andalucia	Spain	38.263	-2.615	RV	RNA-seq, HiC
ES_BH_1412	Liquid nitrogen	10/6/2019	Brenthis hecate	Male	Ablanque	Castille-La Mancha	Spain	40.927	-2.189	RV	Pacbio, WGS
IT_BH_1622	Ethanol	14/7/2013	Brenthis hecate	Female	Borgo Olivi	Treviso	Italy	46.024	12.280	L. Dapporto, R. Vodă	WGS
IT_BH_1623	Ethanol	22/7/2013	Brenthis hecate	Male	Sasso Tetto	Macerata	Italy	43.007	13.232	L. Dapporto	WGS
RS_BH_1628	Ethanol	27/6/2014	Brenthis hecate	Male	Divcibare, Mt. Maljen	NA	Serbia	44.122	20.015	R. Vodă, V. Dincă	WGS
GR_BH_1631	Ethanol	3/7/2014	Brenthis hecate	Female	Granitis	East Macedonia and Thrace	Greece	41.308	23.905	R. Vodă, V. Dincă	WGS
RO_FA_934	Liquid nitrogen	17/7/2018	Fabriciana adippe	Male	Pin1000m, Lupsa, Apuseni Mt.	Alba	Romania	46.416	23.192	KL, RV, Alex Hayward, Dominik R. Laetsch	RNA-seq

Table S2: Maximum composite likelihood parameter estimates for a demographic model describing the divergence history of three *Brenthis* species. Values are given to three significant digits. Lower and upper 95% confidence intervals (CIs) were calculated from parametric bootstrap simulations. For some parameters (*) the point estimate fall outside of the 95% CIs. The → of each m_e parameter denotes the direction of migration backwards in time.

**Parameter**	**Lower 95% CI**	**Point estimate**	**Upper 95% CI**
N_e *daph*	185,000	212,000 *	208,000
N_e *ino*	1,140,000	1,260,000 *	1,230,000
N_e *hec*	1,330,000	1,460,000 *	1,450,000
N_e *daph + ino*	99,500	130,000	859,000
N_e *hec ancestral*	18,800	55,600	123,000
N_e *daph + ino + hec*	1,800,000	2,560,000	4,730,000
Split *daph + ino*	2,360,000	2,790,000	3,000,000
Split *daph + ino + hec*	3,030,000	3,200,000	8,520,000
m_e *daph* →*ino*	1.58×10^{-7}	1.68×10^{-7}	2.16×10^{-7}
m_e *daph* →*hec*	9.42×10^{-9}	1.13×10^{-8}	2.43×10^{-8}
m_e *ino* →*daph*	2.33×10^{-9}	4.48×10^{-9}	1.28×10^{-8}
m_e *ino* →*hec*	6.89×10^{-9}	6.03×10^{-9} *	1.29×10^{-8}
m_e *hec* →*daph*	4.91×10^{-9}	5.26×10^{-9}	9.37×10^{-9}
m_e *hec* →*ino*	1.65×10^{-8}	1.59×10^{-8} *	2.41×10^{-8}
m_e *daph + ino* →*hec ancestral*	5.43×10^{-9}	4.33×10^{-7}	6.51×10^{-7}
m_e *hec ancestral* →*daph + ino*	2.39×10^{-8}	3.53×10^{-7}	5.65×10^{-7}

Table S3: Summary statistics for each species and corresponding parameter estimates under a finite-island model. H is per-4D-site heterozygosity, d_{xy} is pairwise intraspecific 4D site divergence between individuals sampled from different demes in Europe, and W_{roh} is the proportion of 1 Mb windows covered by a ROH. Estimates of the number of demes, N_e and m_e are given to two significant figures.

**Species**	**H**	d_{xy}	W_{roh}	**Demes**	N_e	m_e
B. daphne	0.0044	0.0048	0.0044	20	19,000	1.3×10^{-4}
B. ino	0.010	0.012	0.022	260	3,400	3.9×10^{-4}
B. hecate	0.0098	0.013	0.013	130	6,500	1.4×10^{-4}

References

Baril T, Imrie R, Hayward A. 2021. TobyBaril/EarlGrey: Earl Grey v1.2. Zenodo. https://doi.org/10.5281/zenodo.5718734.

Baril T, Imrie RM, Hayward A. 2022. Earl Grey: a fully automated user-friendly transposable element annotation and analysis pipeline. bioRxiv. Unpublished. doi: 10.1101/2022.06.30.498289.

Barnett DW, Garrison EK, Quinlan AR, Strömberg MP, Marth GT. 2011. BamTools: a C++ API and toolkit for analyzing and managing BAM files. Bioinformatics. 27:1691–1692.

Bisschop G, Lohse K, Setter D. 2021. Sweeps in time: leveraging the joint distribution of branch lengths. Genetics. 219:iyab119.

Buchfink B, Xie C, Huson DH. 2015. Fast and sensitive protein alignment using diamond. Nature Methods. 12:59–60.

Ebdon S, Laetsch DR, Dapporto L, Hayward A, Ritchie MG, Dincă V, Vila R, Lohse K. 2021. The Pleistocene species pump past its prime: Evidence from European butterfly sister species. Molecular Ecology. 30:3575–3589.

Girgis HZ. 2015. Red: an intelligent, rapid, accurate tool for detecting repeats de-novo on the genomic scale. BMC bioinformatics. 16:1–19.

Gremme G, Steinbiss S, Kurtz S. 2013. Genometools: A comprehensive software library for efficient processing of structured genome annotations. IEEE/ACM Transactions on Computational Biology and Bioinformatics. 10:645–656.

Hoff K, Lange S, Lomsadze A, Borodovsky M, Stanke M. 2015. BRAKER1: Unsupervised RNA-Seq-Based Genome Annotation with GeneMark-ET and AUGUSTUS. Bioinformatics. 32:767–769.

Hoff K, Lomsadze A, Borodovsky M, Stanke M. 2019. Whole-Genome Annotation with BRAKER. pp. 65–95 in *Gene Prediction: Methods and Protocols*. edited by Kollmar M. Springer New York.

Kim D, Paggi JM, Park C, Bennett C, Salzberg SL. 2019. Graph-based genome alignment and genotyping with HISAT2 and HISAT-genotype. Nature Biotechnology. 37:907–915.

Li H, Handsaker B, Wysoker A, Fennell T, Ruan J, Homer N, Marth G, Abecasis G, Durbin R, Subgroup GPDP. 2009. The Sequence Alignment/Map format and SAMtools. Bioinformatics. 25:2078–2079.
